# Effective Use of Ketamine-Dexmedetomidine Following Propofol-Induced Hyperlactatemia: A Case Report

**DOI:** 10.7759/cureus.25764

**Published:** 2022-06-08

**Authors:** Alexander Soto-Edwards, Aaron Kawamoto, Austin Peters

**Affiliations:** 1 Department of Anesthesiology and Perioperative Medicine, Oregon Health & Science University (OHSU), Portland, USA; 2 Department of Clinical Neurophysiology, Oregon Health & Science University (OHSU), Portland, USA

**Keywords:** lactate, neuroanesthesia, neuromonitoring, anesthetic infusions, dexmedetomidine, propofol, ketamine, hyperlactatemia

## Abstract

There are limited options for intravenous anesthetics and a lack of available information on the use of ketamine infusion during intracranial surgeries. We present a patient case report of hyperlactatemia during a craniotomy with neuromonitoring while on a propofol infusion with arterial lactate rising from 2.1 mmol/L to a peak of 5.0 mmol/L before reducing to 3.9 mmol/L after the transition to a mixed ketamine and dexmedetomidine infusion in order to maintain neuromonitoring quality and an appropriate depth of anesthesia. No complications were caused by the use of ketamine during this extended neurosurgery case.

## Introduction

Anesthetic management of neurosurgical cases that involve neuromonitoring requires strict maintenance of anesthetic depth, hemodynamic control, surgical field optimization, and rapid wake-up to facilitate neurologic examination. Volatile anesthetic gases interfere with the integrity of neuromonitoring signals [1], so intravenous anesthetics are needed to maintain the patient’s anesthetized state. Disruptions in neuromonitoring integrity put patients at risk of developing post-operative complications [2]. Propofol is essential to modern IV neuroanesthetics. The anesthetic depth can be interpreted through neuromonitoring channels used to monitor surgical integrity [3] and no clear alternative matching propofol’s anesthetizing properties is readily available. We present a case in which a patient undergoing cranial tumor resection developed hyperlactatemia and describe how we approached the transition of anesthetic management intra-operatively. Written consent for this case was obtained from the patient. This manuscript adheres to the applicable EQUATOR guidelines.

## Case presentation

A 43-year-old, 104 kg man underwent a planned right-sided craniotomy for resection of metastatic melanoma metastases to the brain. The patient previously experienced uneventful surgical resections of melanoma lesions on his left arm in 1997 and 1999. Pre-operative imaging identified numerous presumed metastatic melanoma lesions in the patient’s left hilum, pancreatic neck, multiple subcutaneous soft tissue nodules, multiple osseous vertebral lesions, and his right cingulate gyrus. He was otherwise physically fit and active, with minimal symptoms related to his metastatic cancer except for headaches and dizziness, and was not taking any medications. He had no known drug allergies, and his pre-operative examination and laboratory findings were unremarkable.

Induction of anesthesia was performed with 2 mg/kg propofol, 1 mcg/kg fentanyl, 1 mg/kg lidocaine, and 0.6 mg/kg rocuronium (Table 1).

**Table 1:** Timeline of events and arterial blood gas. min: minutes; pH: potential of hydrogen.

Time (nearest 15 min)	Event	pH	Lactate (mmol/L)	PaCO_2_ (mm Hg)	Bicarbonate (mEq/L)
8:00:00 AM	Anesthesia start	–	–	–	–
9:00:00 AM	Initial incision	7.40	2.1	42	26.1
10:00:00 AM	–	7.41	3.0	38	24.3
11:00:00 AM	–	7.46	3.8	30	21.3
11:30:00 AM	–	7.49	5.0	27	20.8
11:45:00 AM	Propofol discontinued. Ketamine and dexmedetomidine infusions initiated	–	–	–	–
12:30:00 PM	–	7.51	4.9	26	21
1:30:00 PM	–	7.48	4.4	29	21.4
1:45:00 PM	Neuromonitoring discontinued. Intravenous anesthetics discontinued	–	–	–	–
2:30:00 PM	–	7.42	3.9	36	22.9
3:00:00 PM	Surgery end	–	–	–	–
3:30:00 PM	Patient extubated. Neurological examination completed.	–	–	–	–

In addition to standard American Society of Anesthesiology (ASA) monitors, an arterial line was placed to monitor PaCO_2_ for hyperventilation brain relaxation, and electrophysiological neuromonitoring was established to monitor somatosensory evoked potentials (SSEPs), motor evoked potentials (MEPs), and electroencephalogram (EEG). Anesthetic maintenance was achieved with partial intravenous anesthetic, administering 100-150 mcg/kg^−^^1 ^min^−1^ of propofol with 0.1-0.3 mcg/kg^−1 ^h^−1^ sufentanil and isoflurane maintained at 0.4% end-tidal concentration. This is a standard anesthetic maintenance mix for this institution when patients require neuromonitoring and paralysis cannot be maintained. Approximately one hour after induction, an arterial blood gas (ABG) demonstrated expected values except for lactate of 2.1 mmol/L (Table 2); no baseline lactate was available for comparison. Routine serial ABGs to titrate PaCO_2_ demonstrated a persistently increasing lactate level, eventually peaking at 5.0 mmol/L about four hours into the surgery.

**Table 2:** Complete arterial blood gas measurements. pH: potential of hydrogen.

Measure	Time						
	9:00 AM	10:00 AM	11:00 AM	11:30 AM	12:30 PM	1:30 PM	2:30 PM
pH	7.40	7.41	7.46	7.49	7.51	7.48	7.42
PO_2_ (mmHg)	126	86	110	96	82	114	158
PCO_2_ (mmHg)	42	38	30	27	26	29	36
Total hemoglobin (g/dL)	14.6	14.4	13.2	13.8	14.2	13.5	13.0
Hematocrit (%)	44.7	44.3	40.6	42.2	43.5	41.3	40.0
Potassium (mmol/L)	4.4	4.4	4.4	4.3	4.1	4.0	4.0
Sodium (mmol/L)	138	135	132	135	139	141	142
Ionized calcium (mmol/L)	1.18	1.18	1.10	1.14	1.13	1.12	1.12
Chloride (mmol/L)	103	102	102	104	106	109	109
Glucose (mg/dL)	116	113	105	115	129	125	121
HCO_3 _(mmol/L)	26.1	24.3	21.3	20.8	21	21.4	22.9
Base excess	1.2	−0.3	−2.4	−2.6	−2.1	−2.0	−1.6
Lactate (mmol/L)	2.1	3.0	3.8	5.0	4.9	4.4	3.9

**Figure 1:**
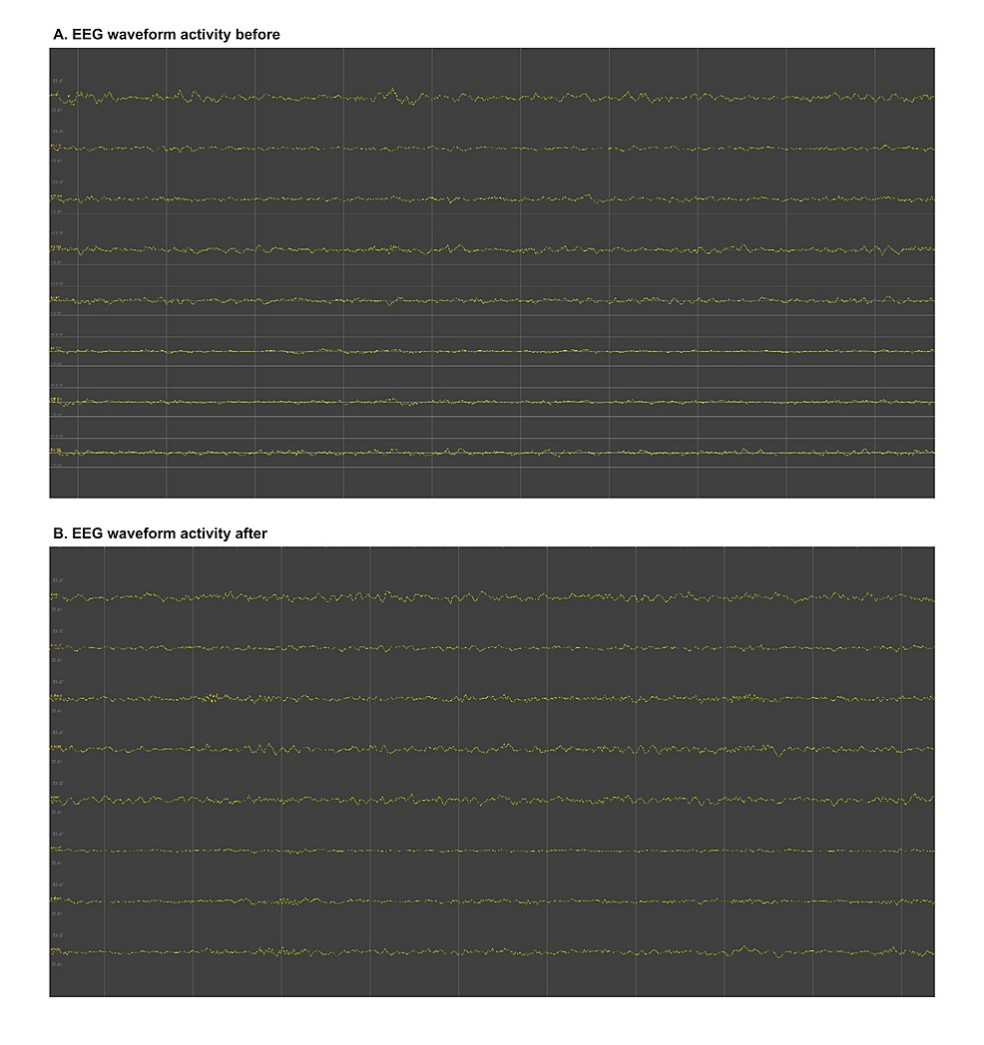
EEG waveform activity before and after the transition of anesthetic infusions. (A) EEG reading from approximately one hour before propofol discontinuation (propofol rate of 100 mcg/kg/h). This activity is of moderate to low amplitude with a dominant delta-theta (4-6 Hz) frequency background, consistent with a continuous sleep state induced by propofol. (B) EEG reading from approximately one hour after conversion to ketamine (8 mg/kg/h) + dexmedetomidine (1 mcg/kg/h) infusions. This activity is of moderate amplitude with a dominant theta frequency (6-7 Hz) background and well-defined intermittent beta activity; theta dominance is characteristic of ketamine’s effect on EEG, and the beta activity reflects dexmedetomidine’s induced sleep-like state.

Explanations for the rising lactate level were investigated. The patient’s blood pressure was sustained within the normal range (105 to 140 systolic and 60 to 80 diastolic) without vasoactive medication support. Fluid input/output was robust throughout the case, with 1.5 liters of urine and two liters of crystalloid administered at the time of peak lactate level was recorded. Over the course of the eight-hour surgery, three liters of urine were produced and five liters of crystalloid were administered, in addition to 1 mg/kg of mannitol for brain relaxation. Both intraoperative triglycerides and creatinine kinase were measured to investigate the possibility of propofol infusion syndrome (PRIS) developing; both laboratory values were within normal limits (122 mg/dL and 34 U/L, respectively). Bleeding was minimal, with approximately 150 mL of total blood loss recorded by the end of the surgery. The patient’s extremities were examined for signs of hypoperfusion or compression, but no evidence of this was seen. He had no known source of infection, and his pre-operative white blood cell count was within normal limits. Neuromonitoring was being utilized, and an EEG examination showed no evidence of seizure or other unexpected activity (Figure 1A). As our patient had no indication of systemic hypoperfusion as a cause of his rising lactate, our consideration then turned to the use of propofol infusion as a possible source of the rising lactate.

In order to maintain neuromonitoring fidelity, a total inhaled anesthetic had to be avoided. In lieu of propofol, we opted to substitute a ketamine infusion at 8 mcg/kg^−1^ min^−^​​​​​​​^1^ with a dexmedetomidine infusion at 1 mcg/kg^−^​​​​​​​^1 ^h^−^​​​​​​​^1^ and continue the sufentanil infusion. After discontinuing propofol, the patient’s lactate declined steadily over the remaining four hours of surgery, with the final intraoperative lactate at 3.9 mmol/L.

Intraoperative neuromonitoring displayed a change in our patient’s EEG from a continuous sleep state indicated by dominant delta-theta frequency with moderate to low amplitudes (Figure 1A, one hour prior to medication transition), to waveforms of dominant theta frequency and intermittent beta activity with moderate amplitudes (Figure 1B, one hour after medication transition). This is consistent with the transition from propofol to a combination of ketamine and dexmedetomidine.

Towards the end of the surgery, when neuromonitoring was no longer indicated, intravenous anesthetics were discontinued, and inhaled isoflurane was used for the remainder of the surgical case. Within 25 minutes of the surgery ending, the patient was safely extubated after meeting all extubation criteria. Within 10 minutes after presenting to the post-anesthesia care unit (PACU), the patient was awake and following directions with normal neurologic function. The patient was observed overnight in the ICU, transferred to a floor room on post-operative day 1, and discharged on post-operative day 3. There were no complications, and he denied any unpleasant experiences or memories.

## Discussion

We present the case of suspected propofol-induced hyperlactatemia during a craniotomy with neuromonitoring requirements and the transition to the use of a ketamine and dexmedetomidine infusion to preserve neuromonitoring quality.

Lactate is an important marker of tissue hypoxia and metabolic dysfunction that is associated with poor outcomes in critically ill and specifically neurosurgical patients [4,5]. Initially, we evaluated intraoperative systemic causes of hyperlactatemia related to end-organ hypoperfusion. There were no signs of active infection (previous laboratory studies were within normal limits and the patient was afebrile). Intraoperative monitoring of cardiac activity was normal; the patient received appropriate intraoperative fluid resuscitation and did not require vasoactive medications while maintaining normal urine output. The patient’s positioning and padding appeared adequate to prevent significant localized ischemic pressure injury. Though ischemia may also be induced by surgical retraction of the brain and surrounding tissue, it is unlikely to cause such a continuous and significant rise in lactate levels. End-tidal carbon dioxide monitoring was not significantly elevated to indicate a hypermetabolic state, and neuromonitoring did not show any evidence of seizure activity (Figure 1A).

After consideration of the possible causes of intraoperative hyperlactatemia, we focused our attention on the propofol infusion as a possible source of our patient’s rising lactate levels. PRIS did not match our clinical scenario as it typically occurs with prolonged infusions greater than 48 hours and with high infusion dosing >4 mg/kg^−1 ^h^−^^1^. In addition to metabolic acidosis, a diagnosis of PRIS requires cardiac abnormalities such as bradycardia or asystole, along with rhabdomyolysis, hyperlipidemia, or myoglobinuria [6,7]. Only hyperlactatemia occurred in our patient; triglycerides and creatinine kinase were within normal limits.

In addition, propofol-induced hyperlactatemia not matching PRIS has been documented in a few other case reports and a limited number of studies [8,9]. The consequences of this isolated hyperlactatemia are less severe than PRIS but have been shown to be associated with prolonged hospitalizations after neurological surgeries, extended intubation, organ failure, and, in some studies, increased mortality [5,10,11]. Although far from being a definitive cause, the relationship between propofol infusion and the patient’s hyperlactatemia was supported by a decline in serum lactate after discontinuation of propofol.

Once we decided propofol was the most likely source of the patient’s hyperlactatemia, and out of concern for potential consequences of continued elevated lactate, an alternative anesthetic agent was needed. The decision of which anesthetic drug to use as an alternative to propofol involves consideration of a drug that is easily titratable, allows reasonably fast emergence, has little impact on increased intracranial pressure (ICP), and has minimal side effects. An etomidate infusion was considered but dismissed out of concern for adrenal suppression with the prolonged infusion. Dexmedetomidine was deemed insufficient to maintain anesthetic depth alone given the need for complete patient immobility and the long context-sensitive half-time, although it does have the possible benefit of lowering ICP [12]. Barbiturates were not available at our institution. Midazolam would not facilitate a fast wake-up after surgery to provide a neurologic exam, and reversal with flumazenil could induce a seizure.

Of the available alternatives, ketamine is the most easily titratable, provides an emergence time similar to propofol, and has the added benefit that it would enhance neuromonitoring signals [13,14].

Potential negative effects of ketamine include increased intracranial pressure (ICP) and cerebral metabolic rate of oxygen (CMRO_2_). Despite the common association of ketamine with elevated ICP and CMRO_2_, these physiologic effects have not been observed in multiple studies. In fact, ketamine administration has been associated with a reduction of ICP and CMRO_2_ in numerous studies [15-17].

A more likely concern from ketamine administration was post-operative delirium or disinhibition interfering with neurologic assessments. Currently, there is no clear guidance on an appropriate ketamine infusion dose to maintain immobility during a general anesthetic case. We opted to use a ketamine dose of 8 mcg/kg^−1 ^min^−1^, providing approximately 50 mg/h of ketamine to our patient. The intravenous anesthetic was supplemented with dexmedetomidine at 1 mcg/kg^−1^ h^−1^ and was then decreased to 0.5 mcg/kg^−1^ h^−1^ over two hours. The observed EEG changes described in Figure 1 are consistent with the transition from propofol to a combination of ketamine and dexmedetomidine: one hour before discontinuing propofol, EEG activity demonstrated a continuous sleep state (delta-theta dominance) associated with propofol infusion; one hour after the transition, EEG demonstrated an induced-sleep-like state with beta activity consistent with dexmedetomidine’s effects and theta dominance consistent with ketamine’s effects. Our patient was extubated in a timely fashion and was able to participate in a neurological examination immediately upon presenting to the post-operative recovery unit. He had an uneventful hospitalization and an efficient discharge home.

## Conclusions

Hyperlactatemia during neurosurgical cases has the potential to negatively affect patient outcomes. When all other possible sources of our patient’s rising lactate were ruled out, we decided to transition from a propofol infusion to a mixed ketamine-dexmedetomidine infusion to maintain the intravenous anesthetic needed to preserve intraoperative neuromonitoring while safely maintaining the patient’s anesthetic depth. This combined ketamine-dexmedetomidine infusion was well tolerated by the patient: lactate trended down, no complications were observed, and a rapid post-operative examination was achieved.

Given the limited alternatives for intravenous anesthetics and the lack of available information on the use of ketamine infusions in complex and delicate neurosurgical scenarios, we hope our experience can provide guidance for other anesthesia providers. Further research is warranted to determine other dosing regimens as well as the impact of ketamine infusion on neurosurgical patient outcomes.
